# COVID-19′s First Wave: Examination of Impact on Food Purchasing Behaviour in the Eurozone

**DOI:** 10.3390/foods10061179

**Published:** 2021-05-24

**Authors:** Belén Gutiérrez-Villar, Rosa Melero-Bolaños, Mariano Carbonero-Ruz

**Affiliations:** 1 Departamento de Gestión Empresarial, Universidad Loyola Andalucía, Escritor Castilla Aguayo 4, 14014 Córdoba, Spain; belengut@uloyola.es; 2 Departamento de Métodos Cuantitativos, Universidad Loyola Andalucía, Escritor Castilla Aguayo 4, 14014 Córdoba, Spain; mcarbonero@uloyola.es

**Keywords:** food prices, COVID-19, Eurozone, Holt–Winters model

## Abstract

COVID-19 has had a negative impact on the living conditions of people in all countries worldwide. With a devastating economic crisis where many families are finding it difficult to pay bills and make ends meet, increases in prices of food basket staples can be very worrying. This study examines the relationship between the incidence of the pandemic during the first wave in 16 Eurozone countries with the variation experienced in food prices. We analysed the harmonised index of consumer food prices (included in HICP) and the classification of the degree of pandemic impact by country, the latter established with the index of deaths provided by the Johns Hopkins Center. The procedure used compared actual food prices during the first wave (March to June 2020) with those foreseeable in the absence of the pandemic. Time series analysis was used, dividing the research period into two phases. In both phases, the Holt–Winters model was applied for estimation and subsequent prediction. After a contrast using Kendall’s tau correlation index, it was concluded that in the countries with the highest death rates during the first wave, there was a higher increase in food prices than in the least affected countries of the Eurozone.

## 1. Introduction

The coronavirus pandemic (COVID-19) is one of the most significant challenges humanity has faced in recent times. Although the disease has affected the entire planet, it has not had the same incidence everywhere and at the same time since it was classified as a pandemic by the World Health Organization (WHO) on 11 March 2020 [1].

Since that fateful moment, humanity has already experienced two peaks in the number of deaths and infections. The first, known as the first wave of the pandemic, lasted from its beginning in March until June 2020; the second wave, whose effects began to be felt at the end of the summer, lasted until the end of 2020, although there have been spikes in cases in January 2021 that warn of the possibility of the beginning of a third wave [2,3].

Today, the coronavirus has affected almost every country on the planet, but its impact during the first wave of the disease was uneven. Among the main factors that, combined, could explain the differences in mortality figures caused by the virus are the speed and restrictive measures imposed by governments, along with factors such as demographics, culture, and the country’s environment [4].

In addition, the suspension of activities and restrictions on movement that many governments have adopted to curb the pandemic have meant a sudden and abrupt change in the lives of citizens and businesses, changes in the structure of demand, and a drastic contraction of the global economy.

According to World Bank forecasts [5], the economic effects of the containment measures undertaken by governments are particularly profound in the countries most affected by the pandemic, leading to significant reductions in employment and incomes. The ILO [6] estimates global income losses over the first three quarters of 2020 (without considering the implementation of income-sustaining measures) at 10.7 per cent compared to the same period in 2019. This reduction in income will not only be projected onto extreme poverty (less than USD 1.90 per day), which is estimated to increase by 20% in 2020 [7], but will also result in new groups facing this situation [8].

From the point of view of the structure of demand, measures relating to the closure of non-essential activities and home confinements have led to a change in the structure of food demand. Thus, closures or capacity constraints in restaurants and catering services in many countries have led to a decrease in the demand for food consumed outside the home [9], while hoarding of products due to panic over possible stock-outs at the beginning of the pandemic, coupled with increased purchases due to the need to eat at home, may have had the opposite effect, i.e., an increase in demand, in this case from households [10,11].

In terms of food availability, governments in most developed countries have placed a high priority on ensuring that food can reach consumers. Overall, it can be argued that, except for occasional moments at the beginning of the COVID-19 pandemic, global staple food supply chains appear to have held up reasonably well, with few instances of substantial supply disruptions, even in countries with strict social distancing requirements [7].

In this dystopian environment, with worsening living conditions for many individuals who have seen their freedoms curtailed across the board, with many jobs lost, and a clear decline in demand for restaurants, bars, and hotels, but with a guaranteed supply of food, some reports raise alarm bells about the rising price of food paid by citizens.

An empirical examination of FAO data by Mogues [12] shows a clear increase in prices paid by the final consumer. In the span of approximately three months between 14 February and 18 May 2020, global average prices for a variety of food commodities increased by between 2% and 9%, with half of the prices tracked increasing by 7% or more. Underlying these averages, however, are large variations between countries.

A macro-survey conducted by the consulting firm IPSOS [13] from 22 May to 5 June among citizens in 26 countries to find out to what extent they perceived a rise in the cost of living in the wake of the first wave of the pandemic points in the same direction. Among the main findings of the survey is that, globally, 60% of respondents believe that the price of food, goods, and services has risen sharply since the arrival of the coronavirus. In particular, the prices of food, groceries, and household products have risen the most for 63% of all respondents. The study also shows differences between countries, with 77% of Belgians and 59% of Germans, to cite the two extremes among European countries.

At the European level, Akter [14] examined the impact of the level of “stay-at-home” restrictions (high or low) and food prices in 31 European countries from January to May 2020. Their findings reveal that the severity of stay-at-home restrictions increased overall food prices by 1% in March 2020 compared to January and February 2020. They continued to increase in countries with high restrictions in April and stabilised in May.

In this environment, it should be a higher priority than ever to study food systems to make them more resilient [15]. Among the many effects that the pandemic shock has caused and the multiple impacts it may have had on food systems, this research focuses only on analysing how food prices have responded during the first wave of COVID-19 in the most important countries. The study focuses on the countries of the Eurozone and the results can be considered by governments to, in other similar situations, take measures aimed at mitigating the short-term inflationary effect that new waves of COVID-19 or other pandemic diseases can cause.

The objective of this research is to study whether there has been a relationship between the price level of food products and the impact of the coronavirus, measured by the death rate, in the countries of the Eurozone during the 1st wave of COVID-19. In the following paragraphs, we review some empirical evidence on the relationship between the incidence of COVID-19 and the evolution of food prices in general and in Europe in particular, during the first wave of COVID-19, which will support the research question on which this paper is built. In subsequent sections, the selected data and the methodology applied are presented and justified, followed by an analysis and a discussion and conclusions.

## 2. Background and Research Hypothesis

### 2.1. Mortality Differences between Countries

On the human level, the lives taken by the pandemic in the first wave rose, according to the data of confirmed deaths as of 30 June 2020, to a total of 511,700 people, of which approximately 23% occurred in Europe [16]. As is well known, the death toll is not evenly distributed among all countries. Table 1 shows the data for the European countries with the largest number of confirmed deaths in the 1st wave of the virus. Europe comprises 50 countries. Among the 10 European countries most affected by the pandemic, eight are part of the Eurozone.

These figures on confirmed deaths are contested and it is very likely that the total number of COVID-19 deaths is higher than confirmed deaths due to differences in the types and number of tests performed and counted by countries, problems in the attribution of cause of death, and delays in reporting [17].

Thus, recent studies on mortality experienced between March and June 2020 compared to the same period in previous years show a clear increase in the number of deaths, suggesting that mortality attributable to COVID-19 during the first wave was higher than officially recognised. Among the EU member states for which data are available, the largest number of additional deaths in 2020 during weeks 10–26 compared to the four-year average from 2016 to 2019 was recorded in Spain (48,000) followed closely by Italy (46,000) and France (30,000) [18].

### 2.2. Evolution of Food Prices

Mead et al. [19], in one of the first studies carried out on the impact of the COVID-19 pandemic on food price indices, studied the volatility in import, export, production, and consumption prices in the months following the onset of the COVID-19 pandemic in the United States, concluding that the shift from institutional and restaurant food consumption to home food consumption created short-term shocks and disturbances in the economy, especially for perishable foods. These shocks affected the economy and affected the prices paid by consumers at the supermarket.

Ebrahimy et al. [20] studied inflation in 107 countries around the world. The only category that showed a noticeable increase in prices at the beginning of the pandemic was food: the price of meat, dairy products, and canned/frozen fruits and vegetables skyrocketed from the beginning of the confinement. The authors studied inflation behaviour for three categories of countries: advanced economies, emerging markets, and low-income countries. In advanced economies, inflation between March and July 2020 was negative in 54.3% of the countries analysed and in 45.9% of emerging market countries and 26.3% of low-income countries. They also related inflation to mitigating policies, finding a low correlation between how strict the mitigating measures were and how high inflation was since March. This seems to be true for both advanced economies and emerging markets. The picture does not change much when looking at individual CPI components rather than the overall index.

**Figure 1:**
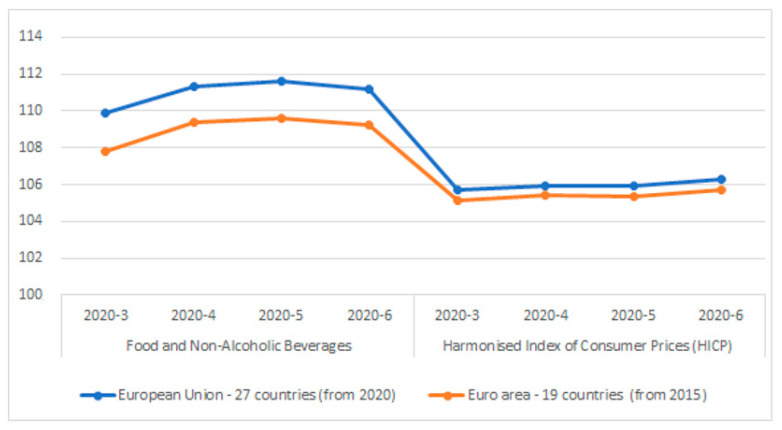
HICP versus food in EU (first wave COVID-19). Elaboration with Eurostat data [21].

If one confines the objective data to the European level and looks at the harmonised index of consumer prices (HICP) published by Eurostat [21], food prices were abnormally high, although a stable trend in headline inflation was observed in the period under study (March–June 2020), both for the EU as a whole and for the Eurozone countries (see Figure 1).

Thus, food price increases in April were strong and reflected an unprecedented combination of supply and demand influences. However, the food HICP in May and June showed signs of normalisation as blockades eased and activity resumed [22].

Similar trends are pointed to by Akter’s [14] study which, after examining the impact of the level of (high or low) “stay-at-home” restrictions and food prices in 31 European countries from January to May 2020, shows that the severity of stay-at-home restrictions increased overall food prices by 1% in March 2020 compared to January and February 2020. They continued to increase in countries with high restrictions in April and stabilised in May.

### 2.3. Research Hypothesis

This paper aims to delve deeper into the impact that the first wave of COVID-19 has had on food prices. As has been explained in the previous sections, the rise in prices is a fact confirmed by economic indicators and the opinions of the public. On the other hand, the figures also show differences between countries in terms of the incidence of the disease during the first wave.

To analyse the impact of the pandemic, we have focused our attention on the Eurozone. There are several reasons that led us to this choice: the high incidence rate of the disease and the priority consideration of health within the scope of action of the European Union, through the implementation of the European Health Union [23], which in practice is leading to a certain homogeneity in the adoption of measures against the disease.

On the other hand, the possibility of examining countries that, in addition to sharing currency, also share economic policy, seems a fundamental aspect for a study that analyses prices, since this common currency avoids effects and conversions related to economic decisions and exchange rates. For this reason, the analyses are confined to countries in the Eurozone.

Therefore, the objective of this research is to study whether there has been a relationship between the price level of food products and the impact of the coronavirus, measured by the death rate, in the countries of the Eurozone during the 1st wave of COVID-19.

Based on this objective, a single analytical hypothesis of difference between groups, stated as alternative hypothesis (H1), is formulated:

**Hypothesis** **1** **(H1).** *In the countries with the highest rate of COVID-19 deaths during the 1st wave, there has been a higher increase in food prices than that experienced in the least affected countries of the Eurozone.*

## 3. Materials and Methods

### 3.1. Confirmed Death Rates in Eurozone Countries

To measure the impact of the disease, the data of officially confirmed deaths in the countries of the Eurozone were selected, in proportion to the size of the country, to better adjust the incidence when making comparisons between countries. For example, if 1000 people died in Malta, with a population of approximately 514,654, that would have a much greater impact than the same number dying in the United States, with a population of 331 million. Therefore, the magnitude used in this study was the confirmed death rate per 100,000 inhabitants. Regarding the size limit, we have chosen to exclude extremely small countries (Malta, Cyprus, and Luxembourg) that are smaller than most of the capitals of the rest of the Eurozone countries.

Therefore, the 19 countries in the Eurozone are reduced to 16, which are those with more than one million inhabitants, with unequal effects of the disease (see Table 2).

We set 30 June 2020 as the date for the collection of data on the incidence of the disease, by which time most countries had already clearly begun to see a drop in the number of new cases or deaths and had begun to relax the restrictive measures adopted to curb the first wave of the pandemic.

### 3.2. The Food Price Index in the Eurozone

Based on previous studies, it can be stated that in EU member states, there are large differences in food prices between member states [20]. For example, in 2019, the price of an equivalent basket of food and non-alcoholic beverages was almost twice as high in the most expensive member state as in the cheapest one [21].

For this paper, the historical series of data for the Food and Non-Alcoholic Beverages category was compiled, taking the harmonised price indices for each of the 16 Eurozone countries with more than one million inhabitants, in the period from January 2013 to June 2020.

**Figure 2:**
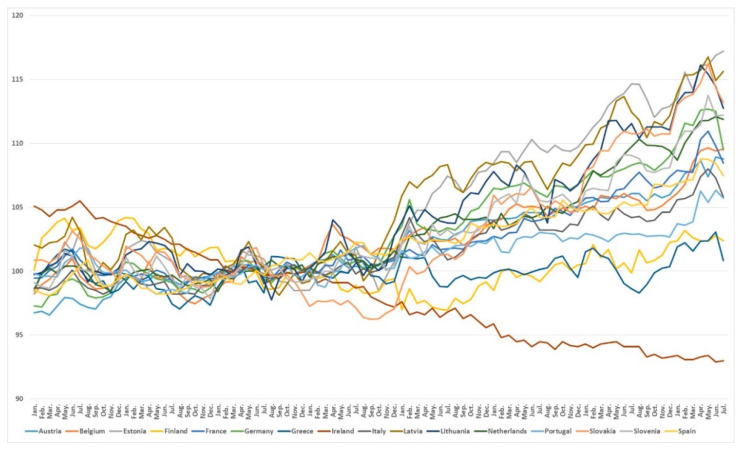
Monthly evolution of harmonised index of consumer prices food and non-alcoholic beverages prices (Eurozone) from January 2013 to July 2020 [21].

When the object of study was limited to European countries with more than one million inhabitants that have the euro as their currency, and time series were constructed by country (see Figure 2), differences between countries were again observed, year by year, and a clear upward trend in food prices was observed in most of the countries studied from 2018 onwards.

### 3.3. Data Analysis

The procedure used in this research was based on comparing actual food prices during the first wave (March to June 2020) with those that would have been expected in the absence of the pandemic. Time series analysis was used, dividing the research period into two phases. In both phases, the Holt–Winter model was applied for estimation and subsequent prediction.

The first phase, called the “learning phase”, consisted of analysing the evolution of the historical series of food category prices in the countries studied, with the intention of predicting their subsequent behaviour. To investigate the price forecasts in each of the countries, the monthly data of the evolution of the HICP historical series have been used, taking January 2013 as the starting month and February 2020 as the final month of learning, the month before the declaration of the pandemic.

This represents a relatively long period of time. Then, if a high level of fit is obtained between the observed and estimated values during this phase, the model predictions for a short number of month (four in this case) should be considered adequate.

To estimate the price series, we have used the Holt–Winters (HW) seasonal algorithm, which is commonly used to capture the effect of seasonality in time series data [24]. This is a triple exponential smoothing procedure, which is especially interesting when, as is our case, we are dealing with short-term forecasting.

The HW algorithm is an iterative method that can be applied to both additive and multiplicative models, with or without stationarity. Among the possible specifications we have chosen, after a descriptive analysis of the price series *(p_t_)*, the additive formulation with a linear trend and annual seasonality is:

$$p_{t}=a_{t}+b_{t}\;t+s_{t}+u_{t}$$

The parameters on which the model depends $\left(a_{t},\;b_{t},\;s_{t}\right)$ were estimated recursively as a function of three quantities, 0≤ α,β, γ≤1, according to the following equations:

$$a_{t}=\alpha \left(p_{t}-s_{t-T}\right)+\left(1-\alpha \right)\left(a_{t-1}+b_{t-1}\right)$$

$$b_{t}=\beta \left(a_{t}-a_{t-1}\right)+\left(1-\beta \right)b_{t-1}$$

$$s_{t}=\gamma \left(p_{t}-a_{t}\right)+\left(1-\gamma \right)s_{t-T}$$

where T is the order of seasonality, the number of time periods until the pattern repeats.

If the series showed a high level of fit, the second phase of the study, called the “prediction phase”, compared the situation of the countries according to the incidence of the epidemic in each of them. If the proposed hypothesis is true, in the countries most affected by the disease, the difference between actual prices during pandemic months (March to June 2020) and those estimated from the model built during the learning phase should be greater.

To obtain the predictions, the equations provided by the Holt–Winters method have been applied during these four months:

$$\hat{p_{f+t}}=a_{f}+b_{f}t+s_{f+t-12},t=1,\;\ldots ,4$$

where the subscript f refers to the situation in February 2020.

Using these predictions, an index of the discrepancy between actual and estimated prices during the period of the first wave was calculated for each country, obtained as the average difference between the four values of each of the two series. A positive value of this measure indicates that prices rose more than they should have in the absence of the pandemic. Conversely, negative values of the index would point to an exceptional decline.

Finally, for testing the hypothesis, the Kendall’s tau index was used to measure the association between the two variables: the incidence of the disease, measured by the death rate, and the price discrepancy index.

Kendall’s tau is a measure of association between two variables. It is like the Pearson’s correlation coefficient but used to compare the ranks (not the values) in a list. The Kendall’s tau coefficient is defined as:

$$\tau =\frac{2\left(C-D\right)}{n\left(n-1\right)}$$

where C and D are the numbers of coincidences and discrepancies in the rank and n is the number of elements in the list.

## 4. Results and Discussion

### 4.1. Learning Phase

To study the past evolution of prices, there are two tasks to be carried out in this first learning phase: the estimation of the series and the measurement of the goodness of fit.

#### 4.1.1. Estimation of the Equation

For the implementation of the HW method, we initialised the parameters a, b, and c estimating the series using a regression model. T = 12 has been taken by assuming that seasonality is annual, as usual with monthly data.

The optimal values of α, β, and γ have been calculated in such a way as to minimise the root mean square error (RMSE) of the learning model predictions. Table 3 and Table 4 show the results for the 16 countries analysed:

The maximum RMSE, obtained in Finland, is 0.806. As the values of all the series are around 100 (the base of the index was set at 100 points for the 2015 average), we can conclude that the RMSE is low in all cases, being a good indication of the achievement of a high degree of accuracy for the model predictions.

Although RMSE is one of the measures usually used as a fit indicator, we consider it convenient to complement it for two reasons: first, the fact that its minimisation has been the guide for estimating the model parameters will tend to reduce its value. Secondly, although, as we have pointed out, its values are very low with respect to the level of the series, there is no standardised reference for its valuation and, therefore, its value is not statistically verifiable.

#### 4.1.2. Goodness of Fit

To measure the quality of the fit, and therefore the reliability of the predictions made with the estimated models, the coefficients of determination between each pair of real and estimated series during the training period (January 2013 to February 2020) have been calculated and statistically contrasted. The results are shown in Table 5:

With the exceptions of Greece and Finland, the coefficient of determination is in all cases above 0.9, indicating a very high level of agreement between the actual and estimated values. Even for these two countries, the value obtained is acceptable. As would be expected under these conditions, all the coefficients obtained are statistically significant, so we can conclude that the values estimated with the models obtained are, in all the countries examined, a good approximation of the true value of the harmonised price index for food.

### 4.2. Prediction Phase

The purpose of this phase is to evaluate the divergence between the prices that occurred between the first wave of COVID-19 (March to June 2020) and the forecasts obtained with the models validated in the training phase during the same period.

**Figure 3:**
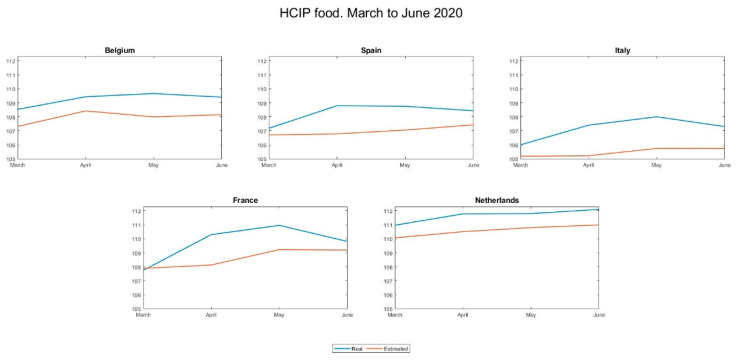
Evolution of the real and estimated price series in the five countries most affected by the pandemic.

**Figure 4:**
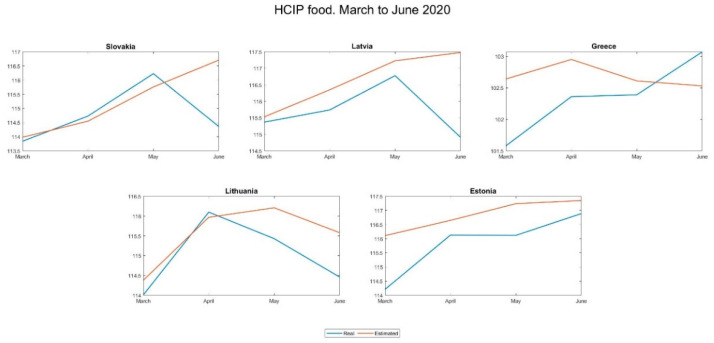
Evolution of the real and estimated price series in the five countries least affected by the pandemic.

Figure 3 and Figure 4 show the evolution of the two-price series (real and estimated) in the five most affected countries and the five least affected by the pandemic:

The differences are manifest and seem to support the hypothesis put forward: in the countries with the highest incidence, prices grew more than expected, while in those less affected this was not the case.

To verify that this impression is correct, we calculated and show in Table 6 the index of discrepancy between actual and estimated prices, as defined in Section 3.3.

This indicator makes it possible to establish a ranking among the countries investigated, headed by Italy, the country with the greatest divergence during the period, and Estonia, which, at the opposite pole, is the country where prices have grown the least with respect to expectations, and even below them.

This ranking, combined with the rate of deaths by COVID-19 as of 30 June 2020, was used to test the validity of the hypothesis of the work. If true, there should be a relationship between the position that countries occupy in both lists. To test whether this is true, we calculated Kendall’s tau correlation index between both rankings, τ = 0.617, and its statistical significance (*p* = 0.001) leads us to the conclusion that both rankings are positively related, in view of the sign of τ, and, therefore, there was a direct relationship, as our hypothesis states, between the incidence of COVID-19 and the rise in food prices during the first wave of the pandemic.

## 5. Conclusions

This research focused on analysing how food prices have responded during the first wave of COVID-19 in the most important countries of the Eurozone. Analysing the relationship between the price level of food products and the impact of the coronavirus, measured by the death rate, in the countries of the Eurozone during the 1st wave of COVID-19, this paper has shown that, during the first wave of the epidemic, there was a greater increase in food prices in the countries most affected by the disease than in those less affected.

Although there are many factors that could be related to this inflationary effect, the choice of countries from the same well-established economic environment (EU-16) and of a certain size (more than one million inhabitants) reduces the list, increasing the probability that the COVID-19 effect is significantly responsible for inflation.

Regarding the methodology, the combined use of a modelling and predictive tool (HW model) and an inferential tool (Kendall’s tau) allows the construction of a hypothetical scenario (estimated prices in the case of no COVID-19) and its statistical comparison with the incidence of the pandemic in each country.

Governments should take these results into account and, in the face of other similar situations, take measures aimed at mitigating the short-term inflationary effect that new waves of COVID-19 or other pandemic diseases may cause. In this sense, it would be advisable for governments to consider measures that strengthen productive capacity on the one hand and help and mitigate the effects suffered by consumers on the other. To date, policymakers have provided massive fiscal support to protect firms, households, and vulnerable populations [1].

On the demand side, social measures have been introduced in the EU to help the most vulnerable consumers, which vary in form and amount depending on the country, but can be summarised as follows: unemployment payments, guaranteed minimum revenue, family support, housing subsidies, and emergency aid [25].

Without making an exhaustive analysis of the measures needed to curb food price increases caused by a pandemic, it seems advisable from the supply side to ensure that they do not occur. Thus, policymakers must act more quickly to weather non-economic disruptions in the supply of goods and services, such as the disruption of international supply chains or reduced labor efficiency due to COVID-19 mobility restriction measures. Other types of fiscal measures, such as the reduction in VAT rates implemented in Germany during the second half of 2020, do not seem to have had the expected effects on consumption [26].

In the case of support to producers, it is necessary to investigate the outcome of the recent package of exceptional measures, including private storage aid of the same product, temporary authorisation to self-organise market measures by operators, and flexibility in the implementation of market support programmes- to further support the agricultural and food sectors most affected by the coronavirus crisis approved by the EU in May 2020 [27].

The findings presented in this document on price increases in the countries most affected by the pandemic refer to the harmonised price index of the Food and Non-Alcoholic Beverages category, without distinguishing between fresh and storable food. It would be interesting to distinguish between them in future research, in order to specify the measures to be taken with each of them.

Finally, it would also be interesting to investigate whether the increase in prices together with the reduction in income due to the reduction in economic activity, which was very pronounced in some groups, has been able to produce changes in the eating habits of consumers.

## Figures and Tables

**Table 1:** Ranking of the 10 European countries with the most cumulative confirmed COVID-19 deaths, 30 June 2020 [16].

Country	Cumulative Confirmed COVID-19 Deaths
United Kingdom	43,880
Italy	34,773
France	29,845
Spain	28,360
Belgium	9747
Netherlands	6132
Sweden	5515
Ireland	1736
Portugal	1576
Luxembourg	110

**Table 2:** COVID-19 deaths per 100,000 inhabitants, 30 June 2020, in 16 countries of the Eurozone ^1^.

Country	Deaths per 100,000 Inhabitants	Country	Deaths per 100,000 Inhabitants
Belgium	84.10	Austria	7.82
Spain	60.64	Finland	5.93
Italy	57.50	Slovenia	5.34
France	45.72	Estonia	5.20
Netherlands	35.78	Lithuania	2.86
Ireland	35.15	Greece	1.84
Portugal	15.45	Latvia	1.59
Germany	10.73	Slovakia	0.51

^1^ Table made with data from Johns Hopkins University [16].

**Table 3:** Optimal values of α, β, and γ ^1^.

Country	α	β	γ	Country	α	β	γ
Austria	1.000	0.000	0.000	Italy	0.891	0.000	1.000
Belgium	0.873	0.000	0.377	Latvia	0.890	0.042	0.649
Estonia	0.842	0.052	0.552	Lithuania	0.986	0.028	1.000
Finland	0.531	0.049	0.006	Netherlands	0.891	0.039	1.000
France	0.781	0.051	1.000	Portugal	0.984	0.000	1.000
Germany	1.000	0.012	0.975	Slovakia	0.952	0.073	1.000
Greece	1.000	0.000	0.000	Slovenia	0.747	0.035	0.801
Ireland	0.802	0.009	0.581	Spain	1.000	0.000	0.998

^1^ Analysis performed with STATA.

**Table 4:** RMSE of the learning model predictions ^1^.

Country	RMSE	Country	RMSE
Austria	0.339	Italy	0.447
Belgium	0.448	Latvia	0.765
Estonia	0.739	Lithuania	0.713
Finland	0.806	Netherlands	0.420
France	0.395	Portugal	0.446
Germany	0.590	Slovakia	0.688
Greece	0.692	Slovenia	0.609
Ireland	0.282	Spain	0.445

^1^ Analysis performed with STATA.

**Table 5:** Coefficients of determination between each pair of real and estimated series during the training period ^1^.

Country	R^2^	*p*-Value	Country	R^2^	*p*-Value
Austria	0.986	0.0000	Italy	0.961	0.0000
Belgium	0.970	0.0000	Latvia	0.972	0.0000
Estonia	0.980	0.0000	Lithuania	0.970	0.0000
Finland	0.824	0.0000	Netherlands	0.985	0.0000
France	0.977	0.0000	Portugal	0.909	0.0000
Germany	0.975	0.0000	Slovakia	0.976	0.0000
Greece	0.693	0.0000	Slovenia	0.968	0.0000
Ireland	0.995	0.0000	Spain	0.970	0.0000

^1^ Analysis performed with STATA.

**Table 6:** Indicator of the discrepancy between actual and estimated prices during the first wave period ^1^.

Country	Index	Country	Index
Austria	0.339	Italy	0.707
Belgium	1.289	Latvia	−0.949
Estonia	−1.001	Lithuania	−0.534
Finland	−0.388	Netherlands	1.068
France	1.097	Portugal	1.580
Germany	0.245	Slovakia	−0.462
Greece	−0.331	Slovenia	0.583
Ireland	0.164	Spain	1.300

^1^ Analysis performed with STATA.

## Data Availability

Population Data: https://ec.europa.eu/eurostat/databrowser/view/DEMO_GIND/default/table?lang=en (accessed on 9 December 2020). Prices: https://ec.europa.eu/eurostat/databrowser/view/PRC_FSC_IDX/default/table (accessed on 9 December 2020). COVID-19 cases: https://github.com/CSSEGISandData/COVID-19 (accessed on 9 December 2020).
